# Dysregulated vitamin D signaling in Hashimoto’s thyroiditis: an integrated transcriptomic study in a Korean cohort

**DOI:** 10.3389/fendo.2025.1666115

**Published:** 2025-10-03

**Authors:** Dong-Woo Lim, Ho-Jung Jeong, Jin Seok Lee, Min-Seo Choi, Sungsoon Fang, Jing-Hua Wang, Hojun Kim, Seok-Mo Kim

**Affiliations:** ^1^ Department of Diagnostics, College of Korean Medicine, Dongguk University, Goyang, Republic of Korea; ^2^ Institute of Korean Medicine, Dongguk University, Goyang, Republic of Korea; ^3^ Ajou University School of Medicine, Department of Surgery, Suwon, Republic of Korea; ^4^ Graduate School of Medical Science, Brain Korea 21 Project, Yonsei University College of Medicine, Seoul, Republic of Korea; ^5^ Department of Biomedical Sciences, Yonsei University College of Medicine, Seoul, Republic of Korea; ^6^ Department of Rehabilitation Medicine, College of Korean Medicine, Dongguk University, Goyang, Republic of Korea; ^7^ Gangnam Severance Hospital, Department of Surgery, Yonsei University College of Medicine, Seoul, Republic of Korea

**Keywords:** Hashimoto’s thyroiditis, vitamin D, RNA-seq, vitamin D regulatory pathway, autoimmune thyroid diseases

## Abstract

**Background:**

Hashimoto’s thyroiditis (HT) is the most common thyroid disease leading to hypothyroidism in developed countries. Recent studies have highlighted vitamin D as a potential risk factor or therapeutic agent for HT owing to its role in modulating immune responses, although concrete evidence has not been presented. This retrospective observational study was conducted to investigate serum vitamin D levels and dysregulation of vitamin D signaling pathways in patients with HT.

**Methods:**

Patients who underwent thyroid surgery for various thyroid neoplasm with or without HT were recruited. We analyzed serum thyroid biomarkers, including serum vitamin D, anti-thyroglobulin (TG) antibody, and anti-thyroid peroxidase (TPO) antibody for patients with HT. Using RNA-seq, the gene expression profile of thyroid tissue and the potential correlation between HT and vitamin D levels or its signaling were investigated.

**Results:**

The serum vitamin D levels were significantly lower in patients with HT. However, vitamin D receptor (VDR) expression and genes involved in vitamin D-associated biological process (BP) were significantly upregulated in the HT group. Visualization of expression profile on Wikipathways revealed multifaceted regulation of vitamin D-related pathways in the HT group. Real-time PCR and immunofluorescence staining confirmed enhanced VDR expression in thyroid tissues from the HT cohort.

**Conclusion:**

Our study presents RNA-seq data acquired from the Korean HT cohort in this study, and highlighted dysregulated vitamin D signaling in thyroid tissues from the HT cohort. Further investigations are needed to elucidate the causal role of vitamin D signaling in the pathogenesis of HT.

## 1 Introduction

Hashimoto’s thyroiditis (HT) is the most common thyroid disease, and cause of hypothyroidism with an incidence of 0.3−1.5 per 1,000 individuals and is steadily increasing (1). HT shows a relatively low prevalence in Asian countries, with an estimated prevalence of around 5.8%, and demonstrates a clear female predominance with a female-to-male ratio of approximately 4:1 (2). HT is characterized by the presence of autoantibodies, including thyroglobulin (TG), thyroid peroxidase (TPO), and the thyrotropin receptor (TSHR), indicating an autoimmune response to self-antigens, leading to chronic inflammation and disruption of thyroid function (3). The risk factors for HT involve a complex interplay between endogenous and exogenous factors (4). Recent studies have suggested potential therapeutic or preventive strategies for managing HT, with vitamin D emerging as a candidate with immunomodulatory effects (4), and being proposed as a potential therapeutic option for HT (5).

Calcitriol, the active form of vitamin D, binds to vitamin D receptors (VDR) to regulate its interaction with the retinoic acid receptor (RXR) (6). RXR and VDR dimers are transported to the nucleus, where they bind to vitamin D-responsive elements (VDRE) and activate the transcription of target genes involved in various physiological processes (7). Vitamin D plays a major role in modulating immune and inflammatory responses by interacting with various immune cells (8). Macrophages are stimulated by calcitriol, which reduces the production of COX-2, and pro-inflammatory factors, while upregulating the production of anti-inflammatory interleukin (IL)-10 (9). Concomitantly, it has been suggested that reduced vitamin D is related to autoantibody production preceding autoimmune diseases due to its effects on B-cell differentiation, maturation, and proliferation (10). More direct evidence has been presented through *in vitro* tests using human B cells for the production of immunoglobulins against pyrogens (11). Vitamin D modulates CD4+ T lymphocyte activity by suppressing Th1 differentiation and promoting regulatory T cell development (12). It also influences dendritic cell-dependent T cell function and mitigates immune tolerance in thyroid tissues under autoimmune conditions (13).

Several studies have reported low levels of vitamin D and its converted forms (calcidiol and calcitriol) in patients with autoimmune diseases (10). Previous epidemiological studies have observed significant correlations among vitamin D deficiency, autoimmune thyroid disease, and elevated antithyroid antibody levels (13). The association between low vitamin D level and the occurrence of autoimmune diseases is well-established; however whether this deficiency is a cause or a consequence of the disease remains unclear (14, 15). Given the proven role of vitamin D in the regulation of immune responses under autoimmune conditions (8), researchers have investigated its direct effect on HT. A prospective randomized controlled clinical trial evaluating vitamin D supplementation in patients with newly diagnosed HT reported significantly attenuated thyroid marker of TPO-antibody (16). However, its direct effects on autoimmune thyroid diseases remain unclear.

To completely elucidate the clinically observed causal relationship between vitamin D deficiency and HT, practical laboratory investigations using patient samples are necessary. However, studies on biochemical changes at the cellular level in clinical thyroid samples from patients with HT are limited in number (17–19). No experimental study has demonstrated vitamin D-related mechanisms underlying the direct correlation between vitamin D or its signaling and HT pathology.

Transcriptomic profiling analysis, including RNA-seq and microarray analysis of the thyroid tissue, is a feasible method to address this problem (20). Using gene expression profiling data combined with bioinformatic tools and analyses, the overall pathophysiological changes in the HT cohort can be explored at the omics level (21). Gene set enrichment analysis (GSEA) can be performed to determine transcriptional activity in the pathway/biological process (BP) of interest in the analysis group (22). WikiPathways is an open-source pathway database that integrates pathway content from other primary pathway databases, including Kyoto Encyclopedia of Genes and Genomes (KEGG), Reactome, and Biopax, and provides broader options for analyzing vitamin D-related pathways (23).

We hypothesized that vitamin D deficiency and altered vitamin D signaling in thyroid tissue are associated with the development of HT. In this retrospective study, we investigated the potential relationship between vitamin D and HT by analyzing gene expression profiles obtained from thyroid tissue RNA-seq in Korean population. Using bioinformatic approaches, we mapped the gene expression profiles of patients with HT in various vitamin D-related wikipathways. Additionally, we validated the results using tissue histology and qPCR to confirm our hypothesis. This research will be useful for elucidating the dysregulation of the vitamin D signaling pathway in autoimmune thyroiditis and highlighting its potential as a therapeutic target. Our findings may also provide a molecular basis for considering vitamin D supplementation as a supportive strategy in HT management.

## 2 Methods

### 2.1 Study subjects and thyroid tissue collection

**Figure 1:**
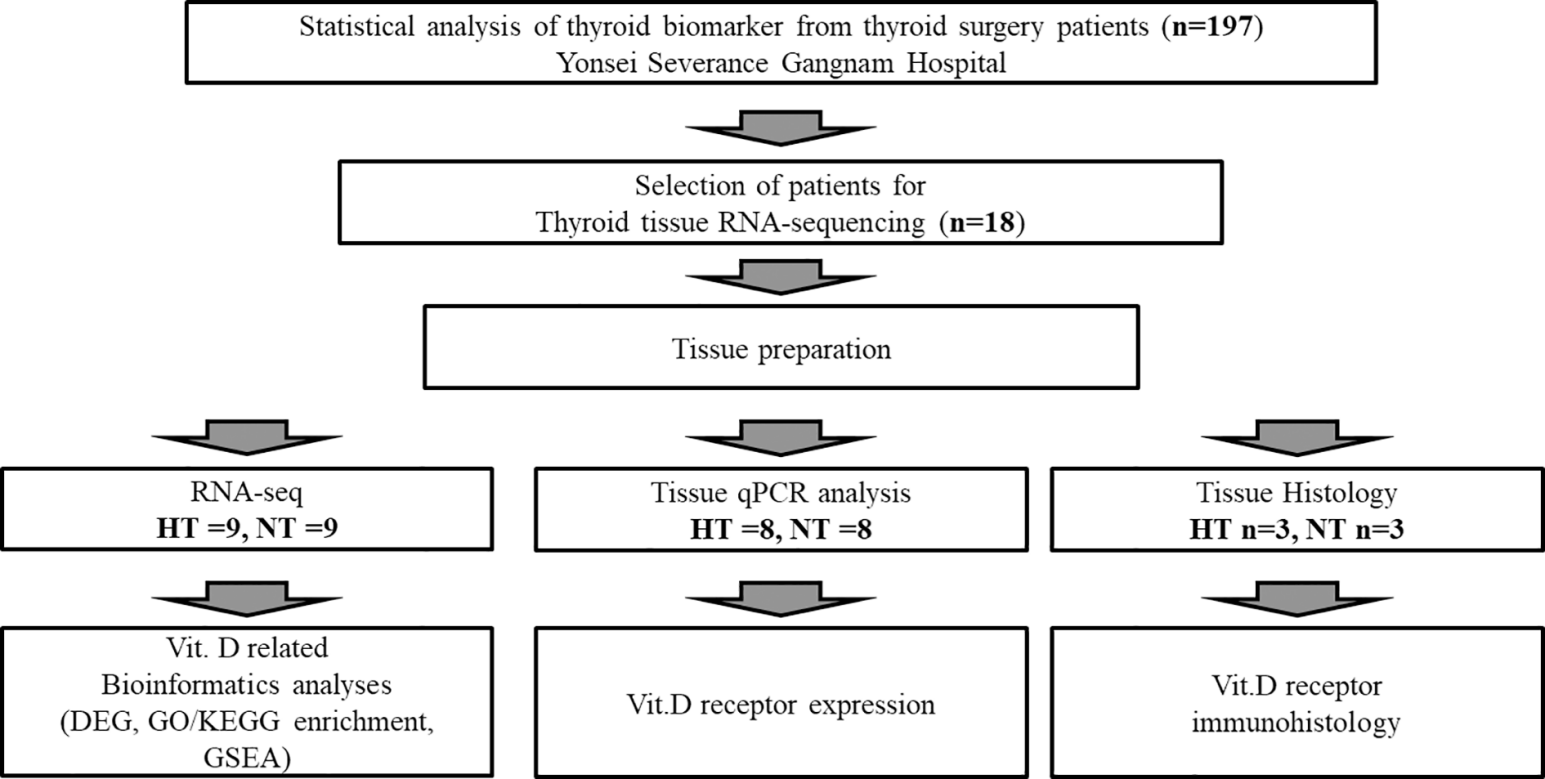
Flowchart showing the overall study design.

This study was conducted in accordance with the Institutional Review Board (IRB) of Yonsei Severance Gangnam Hospital (IRB approval number: 3-2023-0068). This study was conducted in accordance with the Declaration of Helsinki (revised in 2013) and local regulations. The participants were provided with information on the study objectives and procedures. Informed consent was obtained from all participants prior to the study. Patients recruited between July 5, 2023, and December 6, 2024, who were eligible for surgery for various thyroid neoplasms—including papillary thyroid carcinoma (PTC), follicular adenoma, parathyroid adenoma, non-invasive follicular thyroid neoplasm with papillary-like nuclear features, medullary thyroid carcinoma, anaplastic thyroid carcinoma, and adenomatous hyperplasia—were diagnosed based on radiologic findings and histopathologic analysis of tissue obtained through fine-needle aspiration (FNA). For the comparison of clinical features, only female patients with a confirmed histopathological diagnosis of HT were included in the HT group. The use of vitamin D supplements and L-thyroxine therapy was not assessed at baseline. A flowchart of the study design is shown in **Figure 1**. Thyroid tissue collected after partial and total thyroidectomy surgery were transferred and stored at -80 °C. The tissues were used for RNA sequencing, histological analysis, and real-time polymerase chain reaction (PCR) of vitamin D-related markers.

### 2.2 Group assignment for clinical parameters

A total of 197 patients were included in this study. Patients diagnosed with Hashimoto’s thyroiditis were assigned to the HT group (n=87) and those without Hashimoto’s thyroiditis (n=110) were assigned to the NT (normal thyroid) group. Only female patients were included in the clinical comparison to reduce sex-based variability, as female predominance is well-established in HT. No formal power calculation was performed, as this study was retrospective and the available sample size was determined by pre-existing clinical data and biospecimen availability. Linear regression of the correlation between the vitamin D and serum TG levels of the patient was visualized in R studio using the ggplot2 package. Density and histogram plots were used to describe the clinical characteristics of two groups (HT and NT) and the distribution of TG antibody levels in each group. A scatter plot with a linear regression of the two groups was created to describe the correlation between the two variables (TG and serum vitamin D levels).

### 2.3 Differentially expressed genes selection and bioinformatic analysis

A total of 18 tissues from PTC patients, with or without HT, were recruited for the analysis of RNA sequencing. The detailed processes for thyroid RNA sequencing and analysis of gene expression level are described in supplemental information. The generated RNA-seq dataset has been deposited at the Gene Expression Omnibus (GEO) under accession number of GSE 286332. The data quality check and normalization were performed by Macrogen Inc. (Seoul, South Korea), following a standardized protocol. DEG analysis was performed using the DESeq2 package. Statistical significance was determined based on adjusted *p-*values and log2 fold changes. The false discovery rate (FDR) was controlled by adjusting the *p*-values using the Benjamini–Hochberg algorithm. DEGs were selected using a threshold of both adjusted *p-*value < 0.05 and |log2 fold change| > 1.

Enrichment analysis and GSEA for significant BP terms and KEGG pathway was performed using on gProfiler (https://biit.cs.ut.ee/gprofiler/orth) or clusterprofiler with annotation package in R. Using gseGO and gseKEGG function, the package analyze all ranked genes with fold change to output BP terms and KEGG pathways with their adjusted p-value and normalized enriched score (NES). Given the limited sample size, the RNA-seq analysis was designed as an exploratory investigation to identify potential differentially expressed genes and enriched pathways rather than to provide definitive conclusions.

### 2.4 Visualization of gene expression profile on vitamin D-related Wikipathway

Gene expression data were collected from the RNA-seq data. Cytoscape (version 3.10.1) was used to incorporate the gene expression profiles (fold-change) into biological pathways. Among the various biologic pathways listed in WikiPathways, only those directly related to vitamin D signaling and metabolism were included for enrichment analysis, whereas unrelated pathways were excluded. Various vitamin D-related Wikipathways including “Non-genomic actions of 1,25-dihydroxyvitamin D3,” “Vitamin D receptor pathway,” “Vitamin D in inflammatory diseases”, and “Vitamin D metabolism” were loaded using “Wikipathways” Cytoscape app. After importing and attaching gene expression figures as attributes for all genes in the pathways, the gene expression levels were visualized by colorization (red high expression, blue low expression).

### 2.5 Integrative analysis of genes included in vitamin D-related BP terms

Vitamin D-related BP terms were searched using AmiGO2 (https://amigo.geneontology.org/; accessed on October 12, 2024). GO terms were searched using vitamin D as the search query and the filter was set to “Homo sapiens” in the “Organism” field. The resultant BP terms and genes for BPs were identified as gene symbols and further compared with raw expression data acquired from RNA sequencing.

### 2.6 Confirmation of VDR gene expression using real-time PCR

Total RNA was isolated from thyroid tissue using TRIzol reagent (Thermo Fisher Scientific, USA). Reverse transcription was performed using the AccuPower RT PreMix (Bioneer, Daejeon, South Korea) and oligo (dT) 18 primers (Invitrogen, Carlsbad, CA, USA). cDNA amplification was performed using the LightCycler 480 PCR system (Roche, Basel, Switzerland). PCR was performed by following the steps; an initial denaturation step (95 ˚C for 10 min), 45 amplification cycles (denaturation at 95 ˚C for 10 s, annealing at 56 ˚C (*VDR*) and 62 ˚C (*GAPDH*) for 20 s, and extension at 72 ˚C for 30 s). Relative expression levels were calculated by dividing gene Ct values by that of *GAPDH*. Primer sequences of human vitamin D receptor (*VDR*) and *GAPDH*: human *VDR* F 5’-CTGACCCTGGAGACTTTGAC-3’, R 5’-TTCCTCTGCACTTCCTCATC-3’; human *GAPDH* F 5’-GGCCTCCAAGGAGTAAGACC-3’, R 5’-AGGGGTCTACATGGCAACTG-3’.

### 2.7 Histopathological and immunofluorescence staining

Human thyroid tissues fixed in formaldehyde solution (JUNSEI, Kyoto, Japan) were embedded in paraffin and sectioned into 5-μm slices using a Leica microtome (RM2235, Nussloch, Germany). For hematoxylin and eosin (H&E) staining, sections were deparaffinized in xylene, rehydrated through a graded ethanol series, and stained with hematoxylin to visualize nuclei. After rinsing with tap water, the sections were briefly immersed in an eosin solution to stain the cytoplasm and extracellular matrix. For immunofluorescence staining, the sections were deparaffinized, rehydrated, and subjected to antigen retrieval by heating in citrate buffer at 100 °C for 10 min. They were then incubated with a blocking solution containing 2% BSA, 2% goat serum, and 0.1% Triton X-100 in PBS for 30 min. Further, the slides were incubated overnight with a monoclonal VDR (D-6) antibody (1:100; SANTA CRUZ, Catalog #sc-13133, California, USA). Following PBS washes, the sections were counterstained with DAPI (100 μg/mL) to label the nuclei. Finally, all sections were mounted using the VectaMount medium (Vector Laboratories, Burlingame, CA, USA). Stained slides were examined and photographed using a BX53 microscope (Olympus, Tokyo, Japan) equipped with a DP73 camera (Olympus).

### 2.8 Statistical analysis

Statistical differences in clinical characteristics and *VDR* gene expression between the groups were analyzed using an unpaired Student’s *t*-test (two-tailed). Results are presented as means ± standard deviations (SDs), and a *p*-value < 0.05 was considered statistically significant. All statistical analyses were performed using the GraphPad Prism software (version 5.0; GraphPad Software, La Jolla, CA, USA). Linear regression models were constructed using the lm() function in R, and significance was assessed at a *p*-value < 0.05. Post−hoc power was computed in G*Power (version 3.1.9.7) for the independent−samples t−test; standardized effect sizes (Cohen’s d) and 95% CIs were reported.

## 3 Results

### 3.1 Baseline characteristics of the total study cohort

**Figure 2:**
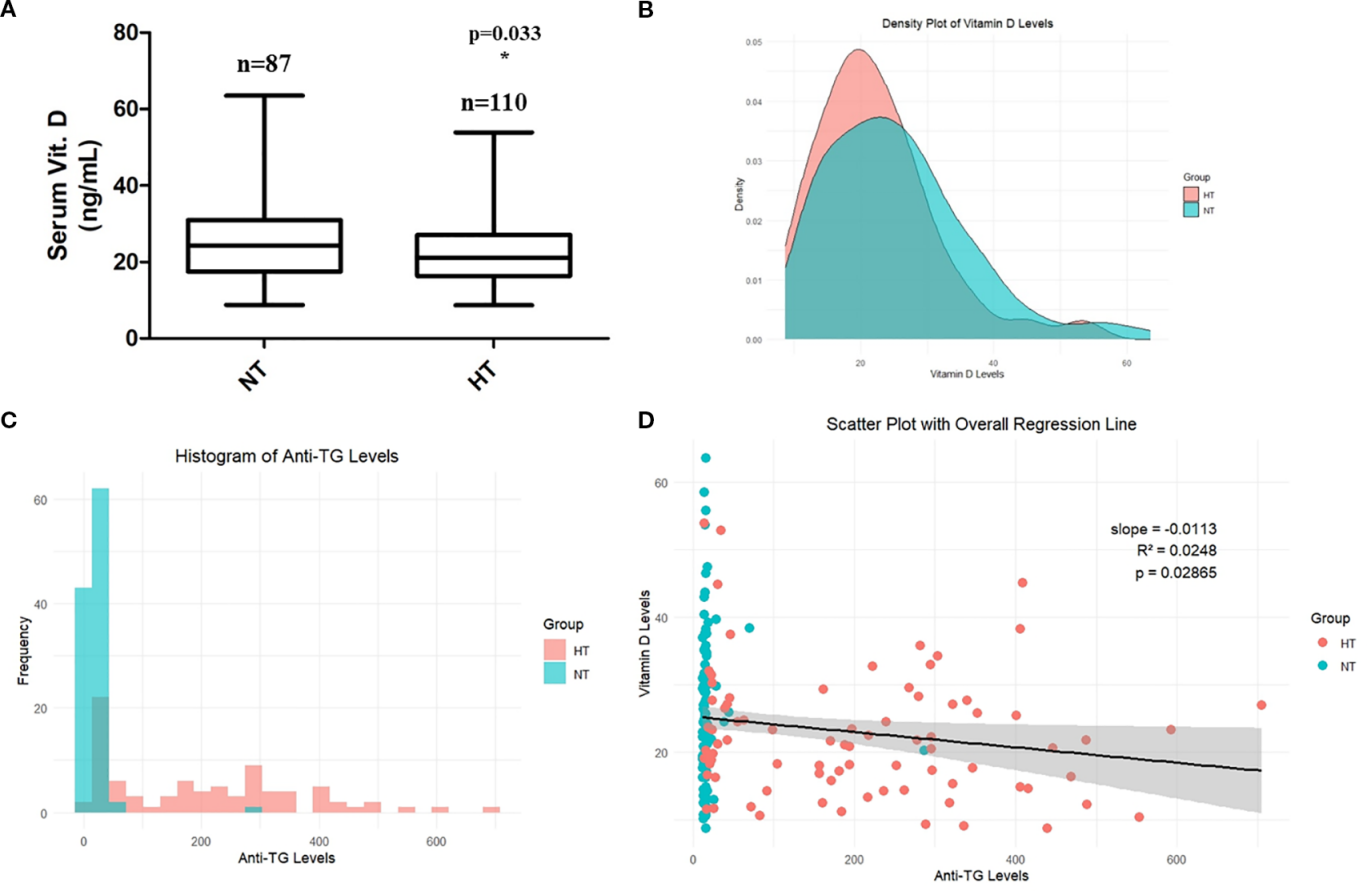
**(A)** Comparison of serum vitamin D levels. **(B)** Correlation between serum anti-thyroglobulin levels and vitamin D levels. **(C)** Histogram showing serum TG levels in NT and HT groups. **(D)** Scatter plot showing linear regression between vitamin D levels and anti-TG levels in NT and HT groups. Data are shown as Mean ± SD. * *p* < 0.05 as compared to control (NT). HT, Hashimoto’s thyroiditis group, NT, normal thyroid group.

The clinical features of the 197 patients are presented in **Table 1**. No significant differences were observed in patient age (years) and serum T4, TSH, and PTH levels (*p* > 0.05), while anti-TG and anti-TPO antibody levels, which are a major clinical feature of HT, were significantly increased in the HT group (*p* < 0.05). Moreover, serum vitamin D levels were significantly lower in the HT group (25.57 ± 10.89 vs. 22.48 ± 9.22 ng/mL, mean difference = 3.09 ng/mL, 95% CI: 0.26–5.92, *p* = 0.033) (**Table 1**, **Figure 2A**). Post−hoc power was 0.556 (α = 0.05). The correlation between anti-TG antibody levels and vitamin D levels was also investigated. The density plot of vitamin D levels of patients from HT and NT showed a difference in distribution between the two groups (**Figure 2B**). Histograms of anti-TG levels showed distinct differences in distribution of anti-TG levels in each group (**Figure 2C**). In addition, a scatter plot with linear regression between the two parameters (vitamin D and anti-TG levels) is displayed (**Figure 2D**). Correlation analysis revealed a statistically significant negative association between thyroglobulin (TG) levels and vitamin D levels in both the HT and NT groups. The model showed an R² of 0.0248 (*p* = 0.0286), indicating weak explanatory power, however, consistent statistical significance.

**Table 1:** Clinical characteristics of total observed patients by groups.

Patients characteristics	NT (n=110)	HT (n=87)	p-value
Sex (n)			
Male	0	0	–
Female	110	87	–
Age, years	44.5 ± 13.26	43.75 ± 12.65	0.685
Anti-TG Ab (IU/mL)	18.82 ± 26.69	234.07 ± 354.36	2.32E-07
Anti-TPO Ab (IU/mL)	10.8 ± 3.59	38.60 ± 46.05	0.0292
Serum T4 (ng/dL)	1.28 ± 0.16	1.30 ± 0.23	0.450
Serum TSH (mIU/mL)	2.47 ± 5.73	3.37 ± 8.90	0.413
Serum PTH (pg/mL)	39.78 ± 15.34	38.57 ± 14.69	0.574
Serum Vit.D (ng/mL)	25.57 ± 10.89	22.48 ± 9.22	0.033

Data are shown as Mean ± SD. HT, Hashimoto’s thyroiditis group; NT, normal group without Hashimoto’s thyroiditis. TG, thyroglobulin; TPO, thyroid peroxidase; T4, thyroxine; TSH, thyroid stimulating hormone; PTH, parathyroid hormone.

(-), p value: not indicated.

While both anti-TG and anti-TPO antibodies are important markers of HT, anti-TG was selected for individual-level correlation analysis (scatter plot) with vitamin D due to its complete availability across the cohort and its higher specificity with HT in certain tissue-based contexts. Anti-TPO data were unavailable for a subset of patients and were therefore not included in individual-level visualizations (scatter plot).

### 3.2 Characteristics of the study cohort for RNA-seq analysis and RNA-seq quality control and data normalization

**Supplementary Table 1** shows clinical characteristics of patients whose thyroid tissue were used for RNA-sequencing. **Supplementary Figure 1** represents data for quality control and data normalization. The detailed description for each figure can be found in **Supplementary File**.

### 3.3 DEG-based GO and KEGG enrichment analysis represents general phenotype of HT

**Supplementary Table 2** shows the top 12 upregulated and downregulated DEGs, respectively, arranged by their Log_2_(FC). *MS4A1*, *P2RX5*, *BLK*, *NIBAN3*, and *POU2AF1* are presented as the top five upregulated genes. *IFI6*, *SLC6A15*, *MYOC*, *IFIT1*, and *PRKG2* are presented as top five downregulated genes.

**Figure 3:**
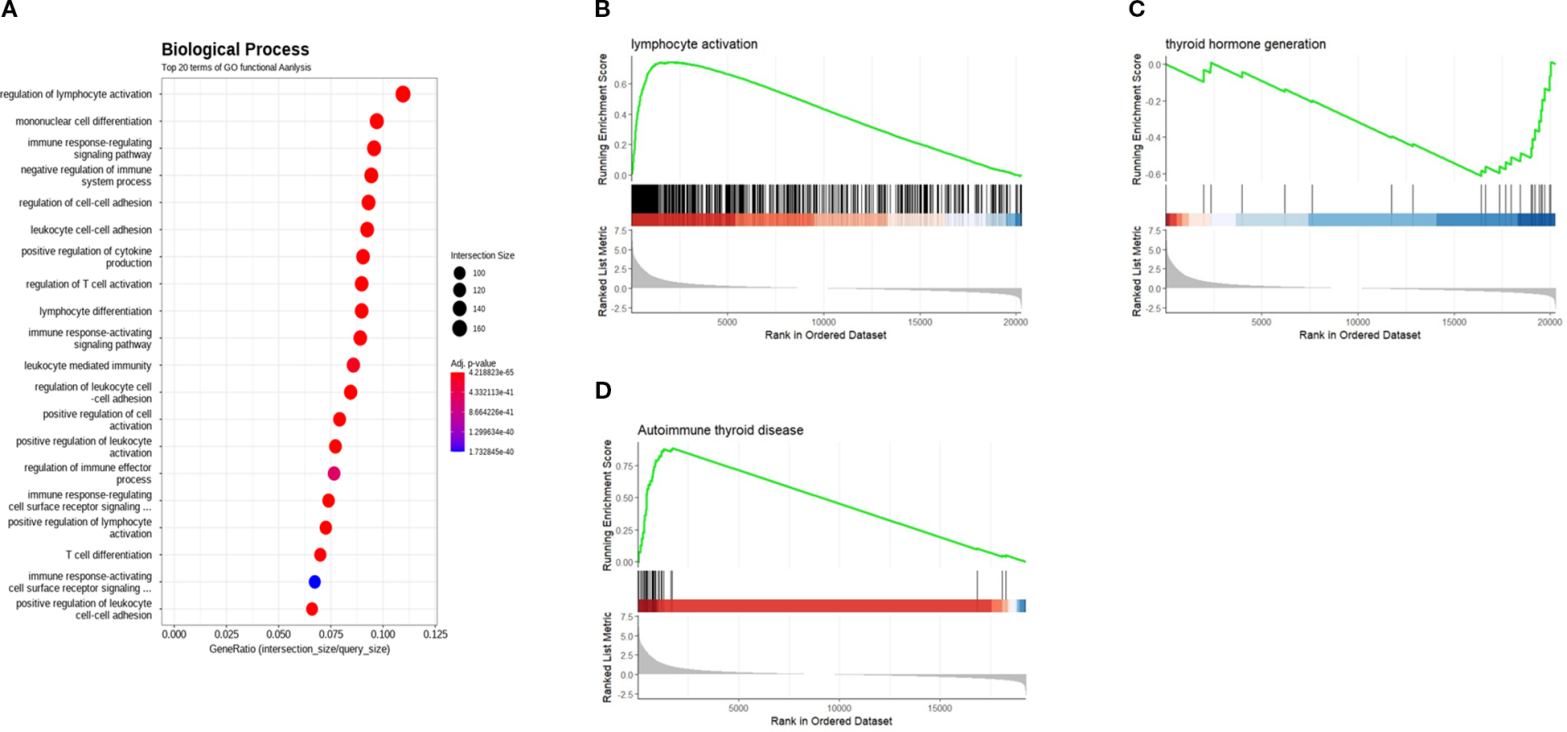
**(A)** Gene ontology (GO) term enrichment analysis from RNA-seq analysis data of the thyroid patients. Biological process (BP) gene set enrichment analysis (GSEA) plot created using the relative gene expression profiles in HT as compared to NT. Significant BP and KEGG GSEA plot of **(B)** Lymphocyte activation and **(C)** thyroid hormone generation. **(D)** GSEA of significant KEGG pathway (autoimmune thyroid disease) in HT. HT, Hashimoto’s thyroiditis group, NT, normal thyroid group.

Furthermore, when enriched BP terms were analyzed with DEGs, T-cell mediated immune related BP terms especially specified in autoimmune thyroid diseases were suggested, including “regulation of lymphocyte activation,” “regulation of T cell activation,” “mononuclear cell differentiation,” and “immune response-regulating signaling pathway” (**Supplementary Table 3**). Furthermore, when enriched BP terms were analyzed with the DEGs, T-cell mediated immune related BP terms especially specified in autoimmune thyroid diseases including ‘regulation of lymphocyte activation’, ‘regulation of T cell activation’, ‘mononuclear cell differentiation’, and ‘immune response-regulating signaling pathway’, ‘regulation of cell-cell adhesion’ were identified (**Figure 3A**). The GSEA plot of significant BP and KEGG pathways revealed the clinical characteristics of HT with definite skewed gene expression profiles related to lymphocyte activation, autoimmune thyroid disease (activated), and thyroid hormone generation (deactivated) (**Figures 3B–D**). A list of significantly upregulated BP terms and KEGG pathways arranged by NES is presented in **Supplementary Table 3**.

### 3.4 Vitamin D-related responses were highly up-regulated in HT

**Figure 4:**
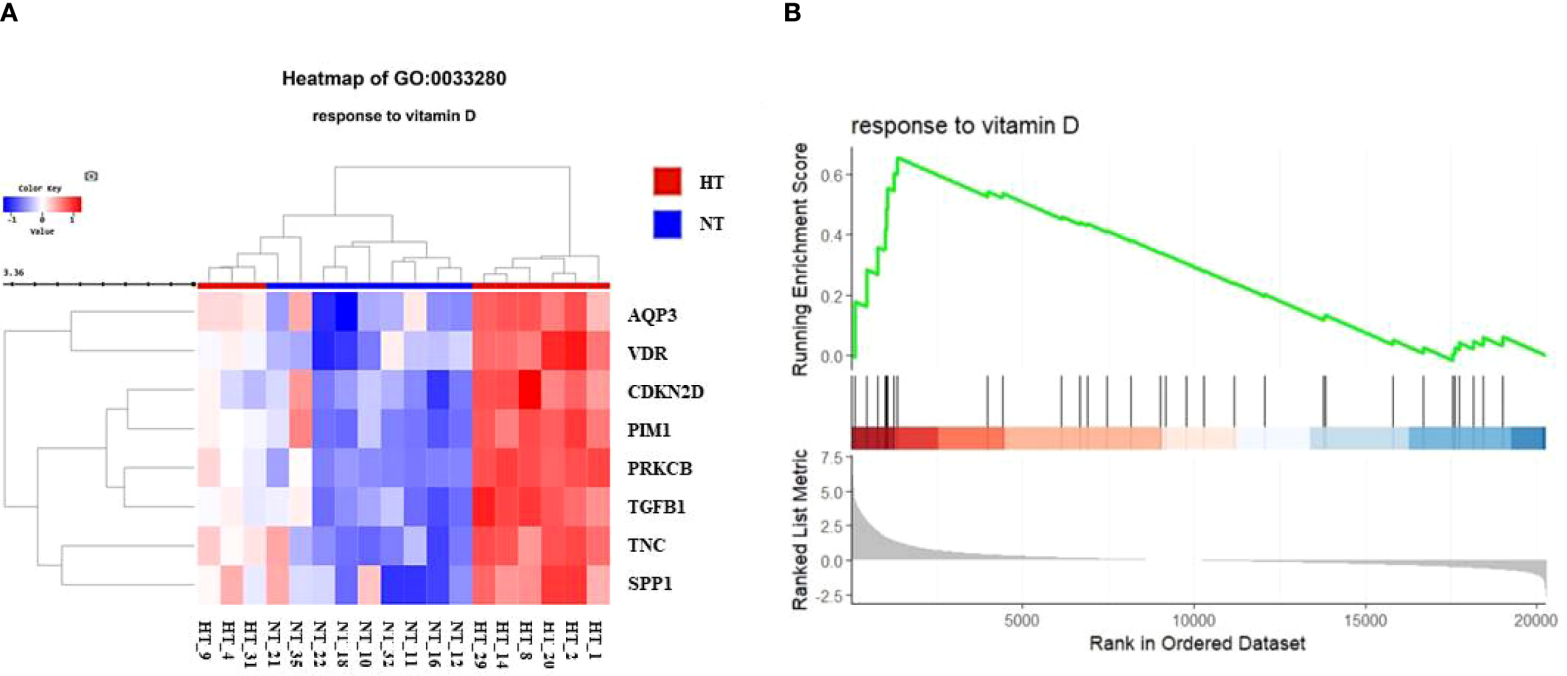
**(A)** Heatmap for comparison of gene expression in vitamin D-related biological process (BP, response to vitamin D) between NT and HT group. HT indicates Hashimoto’s thyroiditis group and NT indicates normal group. **(B)** Gene set enrichment analysis (GSEA) plot showing skewed expression of genes of response to vitamin D in HT group. HT, Hashimoto’s thyroiditis group, NT, normal thyroid group.

We further investigated changes in the vitamin D-related pathway and its signaling in the HT cohort. The response of BP to vitamin D (GO:0033280) was further investigated to confirm the expression levels in each patient (**Figure 4A**). The hierarchical clustering heatmap showed that most individuals with HT had elevated gene expression levels related to the BP term, with isolated individuals per phenotype. The GSEA plot of the BP term “response to vitamin D” revealed activated gene expression profiles (**Figure 4B**).

### 3.5 Visualization of regulation of vitamin D-related pathways via Wikipathway

**Figure 5:**
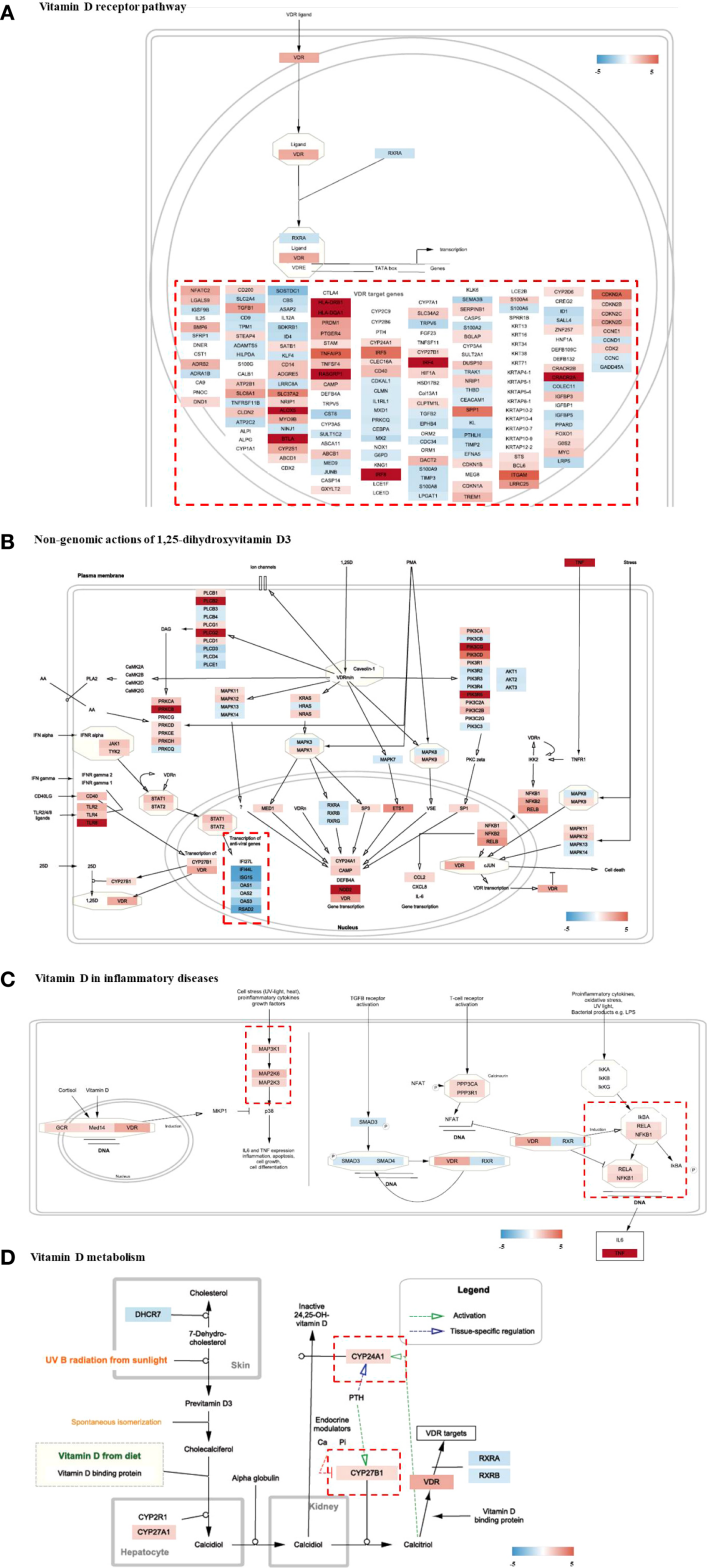
Visualization of gene expression profiling on Vitamin D-related pathways (wikipathways) of HT group as compared to the normal group. **(A)** Vitamin D receptor pathway, **(B)** Non-genomic actions of 1,25-dihydroxyvitamin D3, **(C)** Vitamin D in inflammatory diseases, **(D)** Vitamin D metabolism. HT, Hashimoto’s thyroiditis group.

Genomic expression profiles were further visualized using distinctive colors for genes in each “Wikipathways” (**Figures 5A–D**). In the “vitamin D receptor pathway,” the expression patterns of member genes modulated by the transcription factor of the heterodimeric VDR/RXR complex were individually controlled (**Figure 5A**). Non-classical, non-genomic signaling of vitamin D refers to the rapid activation of intracellular signaling molecules, such as PKC, MAPK, and PI3K/Akt, independent of genomic transcription by the nuclear vitamin D receptor (VDRn). Several genes in signaling pathways were significantly modulated in the HT group (**Figure 5B**). The clustered genes in “transcription of anti-viral genes” (bottom left) showed unanimous downregulation in HT. Pro-inflammatory signaling molecules and major transcriptional factors induced by cellular stresses and cytokines, including MAPK and NF-kB, were concomitantly upregulated (**Figure 5C**). PPP3CA and PPP3R1, which are regulatory subunits of calcineurin and involved in T-cell-mediated immune responses, were upregulated (**Figure 5C**). Analysis of the “metabolism of vitamin D” pathway revealed enhanced expression of genes involved in the metabolism (*CYP24A1*) or biological activation (*CYP27B1*) of vitamin D (**Figure 5D**).

### 3.6 Genes involved in vitamin D-related BPs were modulated in HT

A search query of vitamin D in human BP terms revealed six terms (vitamin D biosynthetic process, vitamin D metabolic process, response to vitamin D, vitamin D receptor signaling pathway, cellular response to vitamin D, and vitamin D catabolic process) from the AmiGO database. Genes involved in vitamin D-related BP terms were investigated based on their expression levels in the RNA-seq expression data (**Table 2**). Of the 18 genes confirmed in our raw expression profile data, eight genes were significantly modified and were all upregulated in patients with HT. None of the genes downregulated in the HT group was statistically significant.

**Table 2:** Gene expression profiles and p-values of vitamin D-related genes extracted from RNA-seq expression data. .

BP term_name	Gene Symbol	Adj. p-value	HT/NT fold change
vitamin D biosynthetic process	CYP2R1	0.211	1.115
	CYP27A1	0.016	1.386
vitamin D metabolic process	PIAS4	0.384	1.042
	CYP27B1	0.810	1.116
response to vitamin D	SFRP1	0.989	-1.005
	SPP1	0.001	4.289
	KL	0.217	-1.282
	AQP3	9.8E-05	3.135
	GDAP1	0.004	-1.433
	TPCN2	0.396	-1.058
	FES	0.843	-1.022
	TNC	2.55E-06	7.318
vitamin D receptor signaling pathway	NCOA3	7.54E-08	2.803
	VDR	7.18E-07	2.634
	PIM1	6.04E-05	3.029
cellular response to vitamin D	PHEX	0.250	-1.646
	BGLAP	0.826	1.080
vitamin D catabolic process	CYP24A1	0.858	1.144

Duplicated genes across the several BPs are allocated in representative BP term. A negative fold change indicates downregulation, where a value of -1 corresponds to a 2-fold decrease in expression. HT, Hashimoto’s thyroiditis group; NT, normal thyroid group without Hashimoto’s thyroiditis. BP, biological process.

### 3.7 Immunohistochemical examination reveals highly expressed VDR in tissue

**Figure 6:**
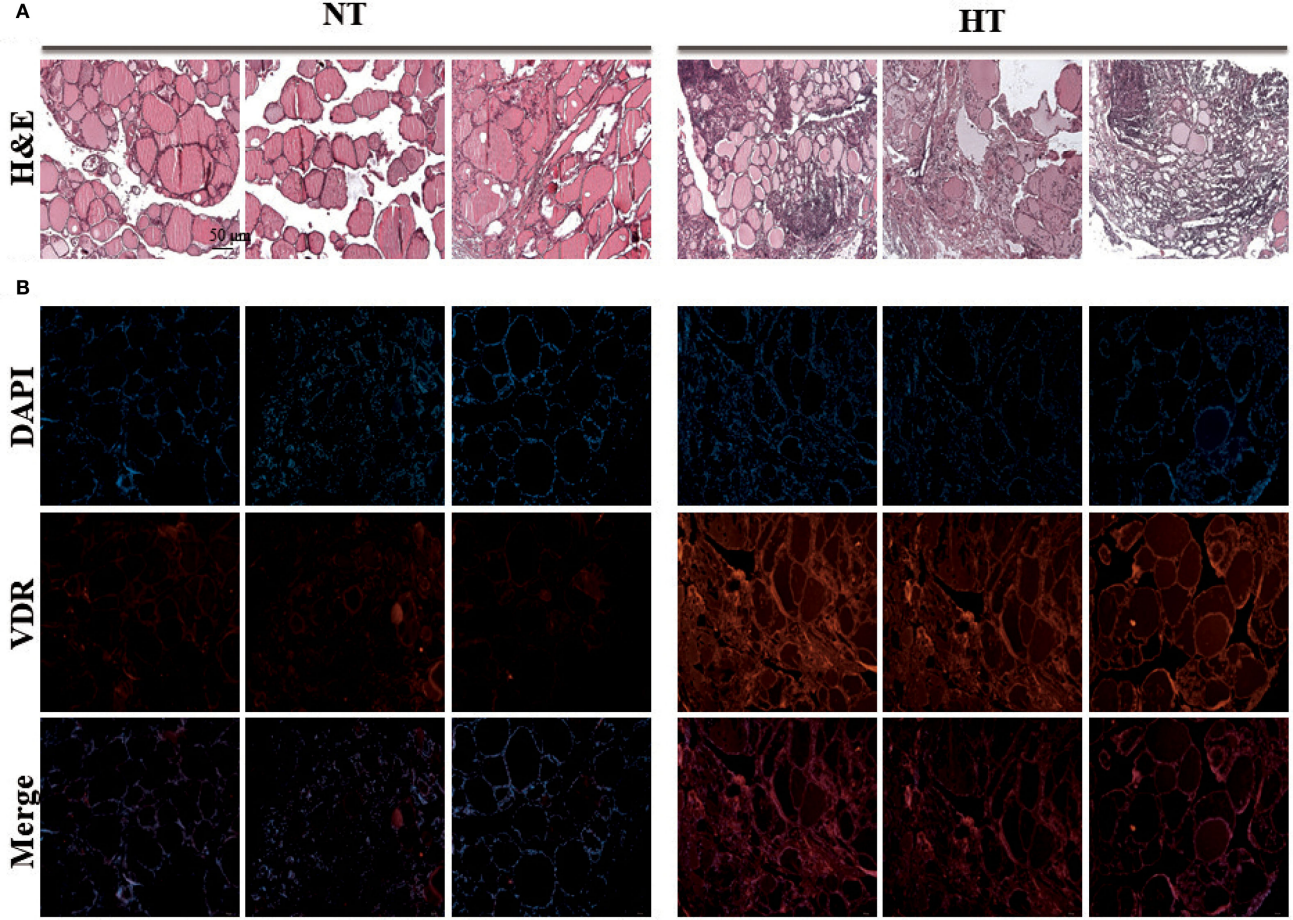
Experimental validation of vitamin D receptor expression by immunostaining analysis. Histology staining was performed with three tissues from different individuals. **(A)** Thyroid tissue stained with H&E staining (H&E stain, ×40). **(B)** VDR protein of thyroid tissue was stained with VDR antibody by Immunofluorescence technique (Immunofluorescence staining, ×40). HT, Hashimoto’s thyroiditis group, NT, normal thyroid group.

To validate these results using vitamin D, we performed real-time PCR and histopathological staining of the thyroid tissue. Real-time PCR of eight samples per group showed significantly upregulated *VDR* gene expression in the HT group, which was consistent with RNA-sequencing data (**Supplementary Figure 2**). Histological examination showed a normal follicle structure without abnormal changes in the NT tissue (left), whereas the HT group showed diffuse boundaries in the structure of the thyroid tissue (right) (**Figure 6A**). Intensified H&E staining demonstrated increased infiltration of immune cells into HT tissue, which is a common clinical feature of Hashimoto’s thyroiditis (**Figure 6A**). In addition, enhanced fluorescence intensity by conjugation of the VDR antibody was demonstrated in HT thyroid tissue (**Figure 6B**).

## 4 Discussion

Several environmental and genomic factors reportedly play a role in the development of HT. In addition, a deep correlation between micro-nutritional factors, including iodine, selenium, and vitamins, and the occurrence of HT is recognized. Moreover, nutritional factors have regional characteristics based on food availability and surrounding environments (24). Vitamin D deficiency in the Korean population is a common problem, prevailing more frequently in women than in men, according to the Korea National Health and Nutrition Examination Survey conducted in 2008 (KNHANES) (25). This phenomenon may have a fundamental impact on autoimmunity-related conditions in the population. The HT cohort showed significantly lower serum vitamin D levels (especially 25(OH) vitamin D) (**Table 1**).

Studies investigating the association between vitamin D levels and autoimmune thyroid diseases have been conducted. A previous study revealed an association between low vitamin D levels and HT in Korean population (26). This study classified patients with HT severity based on the hypothyroidism phenotype: euthyroid (ET), subclinical hypothyroidism (SCH), and overt hypothyroidism (OH). Comparing the vitamin D levels with the non-AITD group, the OH group showed significantly lower vitamin D levels (99.6 ± 53.7 vs 80.1 ± 47.7; *p* = 0.010). Conversely, a phenotype-centered case-control study showed a significantly higher prevalence of vitamin D insufficiency in patients with HT (27). A nationwide survey-based study elucidated the potential link between thyroid autoantibodies or thyroid dysfunction and insufficient vitamin D levels in women (28). A similar (reversal interaction) result between anti-TG levels and vitamin D was observed in our study in all participants (including all NT and HT groups in the statistical analysis). Although the negative correlation between serum vitamin D and anti-TG antibody levels was statistically significant, the very low R² (0.0248) indicates minimal explanatory power. Therefore, this finding should be interpreted with caution and considered hypothesis-generating rather than definitive.

The potential of vitamin D to treat autoimmune thyroiditis was evaluated by investigating the effects of vitamin D supplementation on thyroid antibody and hormone profiles, which resulted in a significant reduction in TG antibody levels (29). The therapeutic potential of vitamin D for the treatment of autoimmune diseases, including multiple sclerosis, rheumatoid arthritis, Crohn’s disease, and systemic lupus erythematosus, was evaluated by reviewing the results from clinical trials and only found inconsistent efficacy by studies (30). Strong evidence supporting the anti-inflammatory effects of vitamin D through the regulation of macrophage and T cell phenotypes may benefit HT patients by alleviating symptoms or, at the very least, preventing disease progression (30).

A meta-analysis of randomized controlled trials performed a combined evaluation of the efficacy of vitamin D treatment on thyroid function and autoimmune markers in patients with HT after a follow-up of three or six months (16). However, the meta-analysis found no significant association between serum vitamin D treatment and the levels of thyroid function and autoantibodies, except for a significant reduction in TPO-Ab levels (16). The inconsistent results of vitamin D supplementation studies highlight the need for a detailed consideration of factors, such as daily dosage, duration, and characteristics of the study cohort. Additionally, tissue-based laboratory analyses should be conducted to closely examine changes within the thyroid.

With the manifestation of the general clinical characteristics of HT by serum markers, activation of immunological responses and changes in thyroid function were significant in the HT group (**Figure 3** and **Supplementary Table 3**). While it was not on the top 12 list of BP terms, “response to vitamin D” was activated with most genes associated with the BP term significantly upregulated in HT (**Supplementary Table 3**). This is the first report to demonstrate activation of the vitamin D signaling pathway in clinical tissue specimens of HT.

As a ligand-induced transcription factor complexed with RXR, VDR modulates the transcription of several genes (31). Dysfunction of the vitamin D receptor (VDR) has been proposed as a key factor in autoimmune diseases, as it degrades innate immune function (32). Previous reports on the correlation between VDR expression and vitamin D levels in pathological status have shown mixed and inconsistent results. An investigation of human VDR expression levels in ageing found no significant association between VDR expression and 25-hydroxyvitamin D (r = 0.002; *p* = 0.994) or 1,25-dihydroxyvitamin D (r = -0.108; *p* = 0.650) levels (33). In a study of patients with prostate cancer, significantly higher VDR expression levels were found in a group with a good prognosis, whereas no significant association was found between circulating vitamin D metabolite levels and VDR protein expression (34). A cohort study on sickle cell anemia reported elevated VDR expression in PBMCs of patients with vitamin D insufficiency (35). However, another study reported decreased VDR expression in T lymphocytes in the HT group, as demonstrated by qPCR analysis (36).

Our study revealed increased VDR expression and its related markers in the HT group. In addition, the expression of VDR-targeted genes showed mixed patterns due to the genomic action of vitamin D (**Figure 5A**). Consistently, reduced vitamin D levels and increased VDR expression were simultaneously observed in HT (**Supplementary Table 1**, **Table 2**), whereas the response to vitamin D was skewed toward activation (**Figures 4A, B**). Marshall’s model suggests that VDR dysfunction can explain the disrupted vitamin D analog levels (low 25-D (inactive form) and high 1,25-D (active form) serum levels) observed in a variety of autoimmune diseases (32). Several hypotheses could explain this paradoxical result including local activation of vitamin D by immune cells, ligand-independent VDR activation, and VDR gene polymorphisms that modulate its response to ligands.

In inflammatory diseases, the local levels of active vitamin D (1,25(OH)_2_D) can be excessively elevated by activated macrophages and dendritic cells (37, 38), achieves local regulation of the vitamin D pathway at inflammatory sites by converting the inactivated vitamin D to active form (30). The pro-inflammatory status in our HT cohort was assumed to be due to the increased expression of transcription factors of inflammatory mediators, along with enhanced phospholipase (PLCs) and Protein Kinase C Alpha (PKAs) molecules (**Figure 5B**). In addition, histological examination revealed the diffuse boundary of the thyroid follicle structure in HT, which was oriented by an autoimmune response in the local tissue (**Figure 6A**). However, vitamin D can attenuate viral infection-induced inflammation and TLR3-mediated immune responses (39). Moreover, VDR on the macrophage membrane (VDRm) contributes to cell tolerance to LPS, suggesting the possibility of reducing inflammation through the suppression of innate immune responses.

VDR expression is regulated by LPS, which activates its sensing molecule, a member of the toll-like receptor (TLR) family (40). In monocytes, activated TLR and IFN-α induces autocrine and paracrine production of calcitriol by regulating CYP27B1 (41). In CD4+ T cells, calcitriol inhibits IFN-r production in Th1 cells and IL-4 production during Th17 cell differentiation and activation (8). Inhibition of vitaminD3 metabolism by blocking the CYP24A enzyme can enhance VDR signaling in cell level (42). Two genes participating in vitamin D activation and metabolism (*CYP27B1* and *CYP24A1*) were slightly, however, not significantly, upregulated (**Figure 5D** and **Table 2**). This may be due to the harvested tissue containing not only immune cells but also other types of cells from the thyroid tissue, making expression unclear.

The versatile biological activity of VDR-including genomic, non-genomic, ligand-dependent, and ligand-independent mechanisms- adds complexity to its interpretation (43). The ligand-independent function, similar to that of other nuclear hormone receptors, acts as a transcription regulator, with its specific roles varying across different cell types (44). The unliganded VDR exhibits non-classical actions by directly interacting with transcription factor or intracellular signaling molecules such as MAPK and PI3K which can have significant impact on innate and adaptive immunity (45, 46). Whether the increased vitamin D-related responses observed in the HT group are pathological characteristics or physiological adaptations to the activated immune response remains unclear.

Post-translational modifications (PTM) regulate VDR activity in diverse ways (47). Phosphorylation by PKC-β (Ser51) enhances transcriptional activation (48), while PKA-mediated phosphorylation (Ser182) reduces VDR–RXR interaction (49). In addition, ATM kinase and MAPK pathways (JNK1/p38) further modulate VDR, linking vitamin D signaling to immune regulation (50). These mechanisms highlight the importance of PTMs in shaping VDR function in autoimmune thyroid disease.

Another factor to consider in vitamin D signaling is VDR gene polymorphisms in trial subjects. Vitamin D receptor polymorphisms strongly affect vitamin D signaling (51) and are related to the susceptibility, severity, and development of several diseases (52). However, in some studies, the VDR polymorphism itself did not influence the expression level of VDR in patients with prostate cancer (34). Moreover, VDR polymorphisms were not involved in genetic susceptibility of the HT cohort, even though the prevalence of vitamin D deficiency was evident (70% vs. 18.2% for HT vs. control group; *p* < 0.0001) (53).

A limitation of this study is that we did not measure the active form of vitamin D (1,25(OH)_2_D), which directly reflects local bioavailability and signaling. To fully understand the localized role of vitamin D in HT, the levels of its active form (1,25D) and the differences in the binding affinity of its ligand to VDR should be investigated in thyroid tissue and compared between NT and HT. In addition, further studies are needed to examine VDR polymorphisms to completely understand the etiology, susceptibility, and progression of the disease in relation to vitamin D signaling. Another limitation is that information on vitamin D supplementation was not available. Because supplementation could directly influence serum vitamin D levels, this represents a potential confounder in interpreting our findings (54). Future prospective studies should incorporate detailed records of supplement use to clarify its impact.

Our study included only female patients to reduce variability related to sex hormones and immune responses. However, this design limits the generalizability of our findings to male patients, in whom vitamin D metabolism and immune regulation may differ. Future studies including both sexes are warranted to clarify potential sex-specific differences in the relationship between vitamin D signaling and autoimmune thyroiditis. In addition, because both HT and NT tissues were obtained from patients undergoing surgery for thyroid neoplasms, tumor-related microenvironmental influences on vitamin D signaling could not be completely ruled out. This potential confounder should be considered when interpreting the results. The small RNA-seq sample size limits the statistical power and generalizability, so these results should be considered exploratory and hypothesis-generating pending validation in larger cohorts.

Our study presents the first RNA-seq dataset obtained from a Korean HT cohort, and elucidates the dysregulation of vitamin D and its signaling in HT. Through integrated research, we found that the HT population exhibits upregulated expression of *VDR* and other genes associated with vitamin D-related pathways in the thyroid tissue, despite low vitamin D levels in serum. However, the present study design does not allow for a conclusion as to whether vitamin D deficiency contributes to, or results from, the development of HT. Further studies are warranted to clarify the causal relationship between vitamin D deficiency and the development of HT, and should incorporate functional approaches such as measurement of local 1,25(OH)_2_D levels in thyroid tissue, assessment of VDR binding and transcriptional activity, and analysis of VDR gene polymorphisms.

## Data Availability

The datasets presented in this study can be found in online repositories. The names of the repository/repositories and accession number(s) can be found below: https://www.ncbi.nlm.nih.gov/geo/, GSE286332.
