# Supplementary material for: Dysregulated vitamin D signaling in Hashimoto’s thyroiditis: an integrated transcriptomic study in a Korean cohort
Source: Front Endocrinol (Lausanne). 2025 Oct 3;16:1666115. doi: 10.3389/fendo.2025.1666115 (PMC12532006; doi:10.3389/fendo.2025.1666115)
Supplement: Supplementary file 1 [file DataSheet1.docx]

Supplementary Material

**Supplementary methods**

**1. Characteristics of the study cohort for RNA-seq analysis and RNA-seq quality control and data normalization**

Supplementary Table 1 shows clinical characteristics of patients whose thyroid tissue were used for RNA-sequencing. Supplementary Fig. 1 represents data for quality control and data normalization.

**2. RNA isolation and sequencing**

The thyroid tissues of patients diagnosed with PTC or PTC with HT were analyzed using RNA sequencing. Transcriptome sequencing was performed on nine samples per group. Functional thyroid tissues that were differentiated using microscopy were selected for further analysis. The selected raw thyroid tissue was delivered to Macrogen (Seoul, South Korea) for isolation and purification and sequencing progress.

Total RNA concentration was calculated by Quant-IT RiboGreen (Invitrogen, Carlsbad, CA, USA). To assess the integrity of the total RNA, samples run on the TapeStation RNA screentape (Agilent, Santa Clara, CA, USA). Only high-quality RNA preparations, with RIN greater than 7.0, were used for RNA library construction.

TruSeq stranded mRNA library preparation kit (Illumina, San Diego, CA, USA) was used to construct a library for sequencing analysis. Paired-end (2 x 100 bp) sequencing was performed using an Illumina NovaSeqX platform (Illumina, USA). The sequences were mapped against the known reference genome using the HISAT2 program (v2.1.0), and transcript assembly was done using the StringTie ^29^. A genome sequence reference (Genome Reference Consortium GRCh38) was used as the database for mapping.

**3. Analysis of gene expression level**

The relative abundances of gene were measured in Read Count using StringTie. Statistical analyses were performed to find differentially expressed genes using the estimates of abundances for each gene in samples. Genes with at least one of zero count value were excluded for the analysis. Filtered data were log2 transformed and subjected to RLE (Relative Log Expression) normalization.

The generated RNA-seq dataset has been deposited at the Gene Expression Omnibus (GEO) under accession number of GSE 286332

**4. Differentially expressed genes (DEGs) selection and bioinformatic analysis**

A total of 18 tissues from PTC patients, with or without HT, were recruited for the analysis of RNA sequencing. The detailed processes for thyroid RNA sequencing and analysis of gene expression level are described in supplementary information. The generated RNA-seq dataset has been deposited at the Gene Expression Omnibus (GEO) under accession number of GSE 286332. The data quality check and normalization were performed by Macrogen Inc. (Seoul, South Korea), following a standardized protocol. DEG analysis was performed using the DESeq2 package ^29^**.** Statistical significance was determined based on adjusted *p-*values and log2 fold changes. The false discovery rate (FDR) was controlled by adjusting the *p*-values using the Benjamini–Hochberg algorithm. DEGs were selected using a threshold of both adjusted *p-*value < 0.05 and |log2 fold change| > 1.

Enrichment analysis and GSEA for significant BP terms and KEGG pathway was performed using on gProfiler ([**https://biit.cs.ut.ee/gprofiler/orth**](https://biit.cs.ut.ee/gprofiler/orth)) or clusterprofiler with annotation package in R. Using gseGO and gseKEGG function, the package analyze all ranked genes with fold change to output BP terms and KEGG pathways with their adjusted p-value and normalized enriched score (NES).

**4**. **Confirmation of VDR gene expression using real-time PCR**

Total RNA was isolated from thyroid tissue using TRIzol reagent (Thermo Fisher Scientific, USA). Reverse transcription was performed using the AccuPower RT PreMix (Bioneer, Daejeon, South Korea) and oligo (dT) 18 primers (Invitrogen, Carlsbad, CA, USA). cDNA amplification was performed using the LightCycler 480 PCR system (Roche, Basel, Switzerland). PCR was performed by following the steps; an initial denaturation step (95 ˚C for 10 min), 45 amplification cycles (denaturation at 95 ˚C for 10 s, annealing at 56 ˚C (*VDR*) and 62 ˚C (*GAPDH*) for 20 s, and extension at 72 ˚C for 30 s). Relative expression levels were calculated by dividing gene Ct values by that of *GAPDH*. Primer sequences of human vitamin D receptor (*VDR*) and *GAPDH*: human *VDR* F 5’-CTGACCCTGGAGACTTTGAC-3’, R 5’-TTCCTCTGCACTTCCTCATC-3’; human *GAPDH* F 5’-GGCCTCCAAGGAGTAAGACC-3’, R 5’-AGGGGTCTACATGGCAACTG-3’.

**Supplementary results**

**1. Characteristics of the study cohort for RNA-seq analysis**

Supplementary Table 1 shows clinical characteristics of patients whose thyroid tissue were used for RNA-sequencing. Similarly, anti-TG levels were statistically increased in the HT group (*p* = 0.001) whereas other clinical features were not (*p* > 0.05). Vitamin D levels were relatively low in the HT group, however, did not show statistical significance (*p* = 0.096), due to the small population in each group (n = 9).

**2. RNA-seq quality control and data normalization**

Among the 46,427 genes, those with a count value of zero in at least one of the 18 samples were excluded from the analysis. Statistical analyses were conducted on the remaining 20,280 genes, after removing 26,147 genes. Supplementary Fig. 1 presents boxplots that visually represent the distribution of expression values across samples for raw read counts, log2(read count + 1), and values normalized using Relative Log Expression (RLE). Using rlog-transformed values, we assessed the degree of similarity between samples by calculating Pearson's correlation coefficients (Range: -1 ≤ r ≤ 1). A correlation coefficient close to 1 indicates a high similarity between samples. The correlation matrix for all samples is shown in Supplementary Fig. 1B.

A two-dimensional representation was generated based on the first two principal components, reflecting sample-to-sample similarities (Supplementary Fig. 1C). This visualization allows the identification of potential outlier samples and clustering patterns that indicate similar expression profiles among the sample groups. For significant DEGs, a hierarchical clustering analysis (using Euclidean distance and complete linkage) was performed based on the rlog-transformed expression values for each sample and gene (Supplementary Fig. 1D). This analysis grouped samples and genes with similar expression levels into clusters and visualized them accordingly. Supplementary Figure 1E shows the volcano plot generated to depict the results of the differential expression analysis, comparing log2 fold changes in expression between the two groups and their associated *p*-values. The X-axis represents the log2 fold change, whereas the Y-axis shows the -log10-transformed *p*-value, highlighting significantly regulated genes in HT group.

**Supplementary Table**

| Patients  characteristics | NT (n=9) | HT (n=9) | p-value |
| --- | --- | --- | --- |
| Sex (n)  Male  Female | 2  7 | 0  9 | 0.47 |
| Age, years  Mean$\pm$SD | 48.0$\pm11.18$ | 43.22$\pm$10.24 | 0.359 |
| Anti-TG Ab  Mean$\pm$SD | 16.88$\pm$5.50 | 219.32$\pm127.22$ | 0.001 |
| Anti-TPO Ab  Mean$\pm$SD | 11.6$\pm$4.12 | 75.18$\pm61.49$ | 0.081 |
| Serum T4  (ng/dL) | 1.26$\pm$0.16 | 1.38$\pm$0.27 | 0.230 |
| Serum TSH  (mIU/ml) | 1.83$\pm$1.48 | 2.29$\pm$1.11 | 0.460 |
| Serum PTH  (pg/ml) | 37.64$\pm$12.33 | 34.17$\pm$13.88 | 0.582 |
| Serum Vt.D  (ng/ml) | 34.13$\pm$18.04 | 22.63$\pm$4.39 | 0.096 |

**Supplementary Table 1. Clinical characteristics of RNA-seq analysis groups. Data are shown as Mean ± SD.**

|  |  | **Gene name** | **Log_2_(FC)** | **P-value** |
| --- | --- | --- | --- | --- |
| **Up-**  **regulated** | **MS4A1** | membrane spanning 4-domains A1 | 6.415 | 2.558E-12 |
|  | **P2RX5** | purinergic receptor P2X 5 | 6.015 | 7.834E-13 |
|  | **BLK** | BLK proto-oncogene, Src family tyrosine kinase | 5.970 | 5.794E-37 |
|  | **NIBAN3** | niban apoptosis regulator 3 | 5.933 | 9.931E-21 |
|  | **POU2AF1** | POU class 2 homeobox associating factor 1 | 5.573 | 8.452E-25 |
|  | **IGLL5** | immunoglobulin lambda like polypeptide 5 | 5.573 | 4.305E-15 |
|  | **UBD** | ubiquitin D | 5.556 | 1.127E-20 |
|  | **MEF2B** | myocyte enhancer factor 2B | 5.439 | 2.818E-23 |
|  | **CD79B** | CD79b molecule | 5.434 | 2.233E-23 |
|  | **HLA-DOB** | major histocompatibility complex, class II, DO beta | 5.431 | 6.441E-27 |
|  | **PTPRCAP** | protein tyrosine phosphatase receptor type C associated protein | 5.387 | 8.550E-18 |
|  | **IL21R** | interleukin 21 receptor | 5.352 | 6.714E-18 |
| **Down-**  **regulated** | **IFI6** | interferon alpha inducible protein 6 | -2.668 | 1.196E-06 |
|  | **SLC6A15** | solute carrier family 6 member 15 | -2.641 | 7.294E-09 |
|  | **MYOC** | myocilin | -2.590 | 2.128E-05 |
|  | **IFIT1** | interferon induced protein with tetratricopeptide repeats 1 | -2.525 | 4.613E-06 |
|  | **PRKG2** | protein kinase cGMP-dependent 2 | -2.429 | 2.917E-05 |
|  | **TRARG1** | trafficking regulator of GLUT4 (SLC2A4) 1 | -2.385 | 0.003 |
|  | **PLIN4** | perilipin 4 | -2.215 | 5.754E-05 |
|  | **NRG1** | neuregulin 1 | -2.169 | 3.026E-08 |
|  | **IFI44L** | interferon induced protein 44 like | -2.142 | 0.0007 |
|  | **ISG15** | ISG15 ubiquitin like modifier | -2.104 | 0.001 |
|  | **TNN** | tenascin N | -2.085 | 1.370E-05 |
|  | **PCK1** | phosphoenolpyruvate carboxykinase 1 | -2.014 | 0.004 |

**Supplementary Table 2. List of top 15 up- or downregulated differentially expressed genes (protein-coding genes only). Genes with |Log2(FC)| > 1 and p-value < 0.05 were considered as DEGs**

| **Description (BP)** | **Enrichment**  **score** | **NES** | **Adjusted**  **P-value** |
| --- | --- | --- | --- |
| adaptive immune response | 0.838 | 2.478 | < 0.001 |
| plasma membrane invagination | 0.880 | 2.445 | < 0.001 |
| B cell mediated immunity | 0.846 | 2.440 | < 0.000 |
| immunoglobulin mediated immune response | 0.845 | 2.433 | < 0.000 |
| B cell receptor signaling pathway | 0.886 | 2.432 | < 0.000 |
| phagocytosis, engulfment | 0.887 | 2.432 | < 0.000 |
| immunoglobulin production | 0.847 | 2.430 | < 0.000 |
| membrane invagination | 0.868 | 2.429 | < 0.000 |
| antigen receptor-mediated signaling pathway | 0.834 | 2.418 | < 0.000 |
| lymphocyte mediated immunity | 0.818 | 2.400 | < 0.000 |
| adaptive immune response based on somatic recombination of immune receptors built from immunoglobulin superfamily domains | 0.815 | 2.393 | < 0.000 |
| regulation of B cell activation | 0.831 | 2.391 | < 0.000 |
| humoral immune response mediated by circulating immunoglobulin | 0.891 | 2.389 | < 0.000 |
| positive regulation of B cell activation | 0.849 | 2.385 | < 0.000 |
| positive regulation of lymphocyte activation | 0.810 | 2.381 | < 0.000 |
| **Description (KEGG)** | **Enrichment score** | **NES** | **Adjusted**  **P-value** |
| Hematopoietic cell lineage | 0.8338 | 2.2685 | 0.0001 |
| Leishmaniasis | 0.8267 | 2.2222 | 0.0001 |
| Antigen processing and presentation | 0.8333 | 2.2014 | 0.0001 |
| Inflammatory bowel disease | 0.8459 | 2.1878 | 0.0001 |
| Graft-versus-host disease | 0.9094 | 2.1854 | 0.0001 |
| Th17 cell differentiation | 0.7862 | 2.1778 | 0.0001 |
| Rheumatoid arthritis | 0.8060 | 2.1749 | 0.0001 |
| Allograft rejection | 0.9132 | 2.1679 | 0.0001 |
| Th1 and Th2 cell differentiation | 0.7884 | 2.1564 | 0.0001 |
| Intestinal immune network for IgA production | 0.8797 | 2.1525 | 0.0001 |
| Staphylococcus aureus infection | 0.8320 | 2.1517 | 0.0001 |
| Type I diabetes mellitus | 0.8827 | 2.1506 | 0.0001 |
| Natural killer cell mediated cytotoxicity | 0.7751 | 2.1447 | 0.0001 |
| Autoimmune thyroid disease | 0.8826 | 2.1210 | 0.0001 |
| Tuberculosis | 0.7396 | 2.1011 | 0.0001 |

**Supplementary Table 3. Top up-regulated BP and KEGG pathways arranged by NES**

**
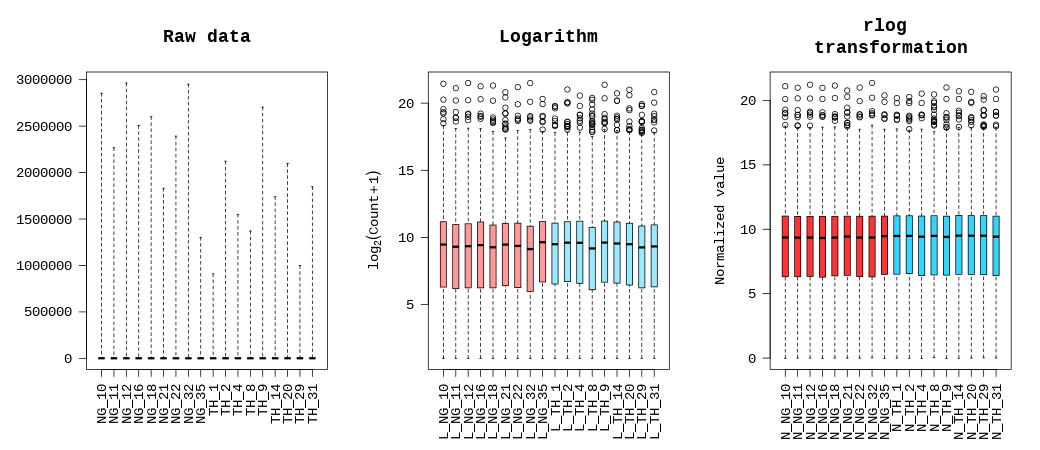

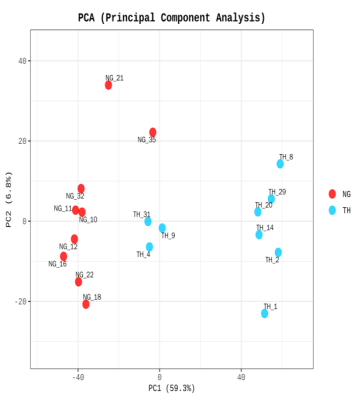

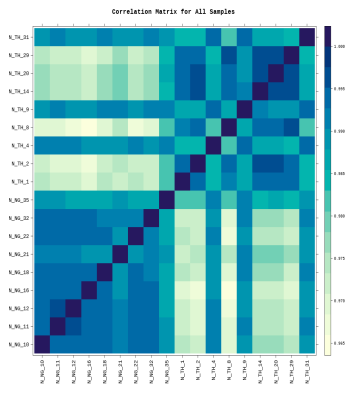

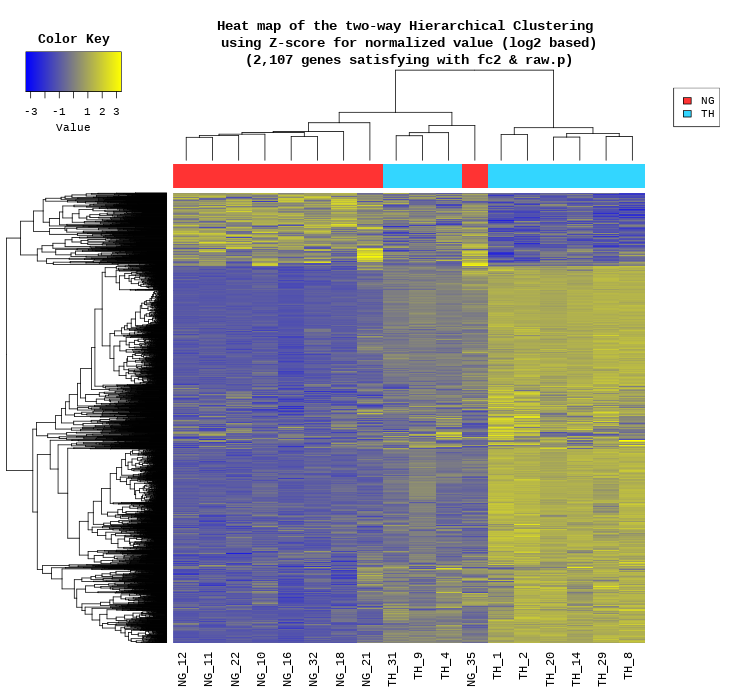

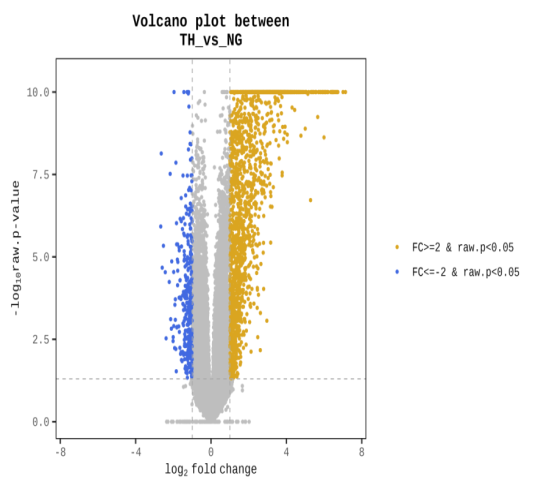
Supplementary Figure**

(A)

(B)

(C)

(D)

(E)

**Supplementary Fig. 1 Quality control data of RNA-seq of the thyroid patients (A) Raw data before and after normalization and log transformation (B) PCA plot of samples analyzed (C) Correlation matrix heatmap for samples (D) Hierarchical clustering of all RNA-seq samples (E) Volcano plot for the screening of up-or down-regulated DEGs**


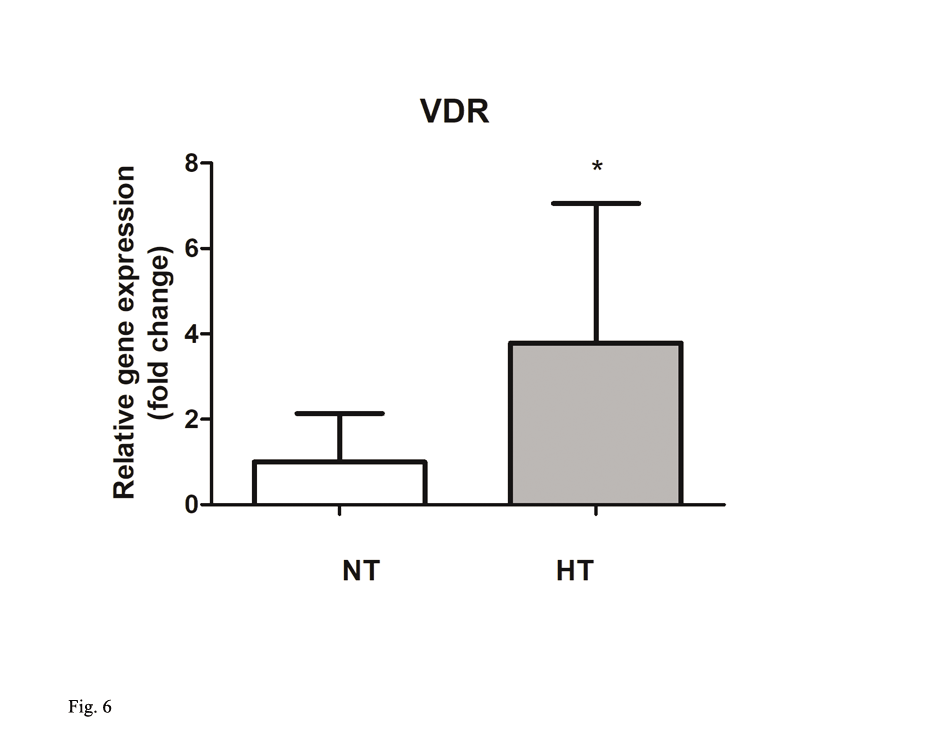


**Supplementary Fig. 2 Experimental validation of vitamin D receptor expression by real-time PCR. Data are shown as Mean ± SD. * p < 0.05 as compared to normal control (NT). HT : Hashimoto’s thyroiditis group (n=8), NT : normal thyroid group (n=8).**
